# Caution needed to avoid empty scale-up of Kangaroo Mother Care in low-income settings

**DOI:** 10.7189/jogh.08.010306

**Published:** 2018-06

**Authors:** Steve Hodgins, Bina Valsangkar, Janna Patterson, Stephen Wall, Joy Riggs-Perla

**Affiliations:** 1 University of Alberta, School of Public Health, Edmonton, Canada; 2 Saving Newborn Lives, Save the Children, Washington, D.C., USA; 3 Bill and Melinda Gates Foundation, Seattle, Washington, USA

Kangaroo Mother Care (KMC) is enjoying growing support from ministries of health, international development partners, health professionals, and families. Recent and established initiatives, such as the *Every Newborn Action Plan*, *Helping Babies Survive*, *KMC Acceleration Partnership*, and *International Network in Kangaroo Mother Care*, combined with a growing evidence base, documentation on scale-up challenges and solutions [1,2], and endorsement by the World Health Organization [3,4], have created favorable conditions for KMC scale-up in many countries. Proliferation of hospital KMC units is often displayed as a proud marker of progress – but how real is the progress we are making? What does it really mean to scale-up KMC?

As a global community, we may be falling short on our efforts to scale effective KMC due to conceptualization of KMC as a *unit* or *place* within the hospital, rather than a set of foundational care practices for premature infants. While there is wide variation in KMC practice in low-income settings, it is all too frequent that KMC may only be initiated a few days before discharge, in a step-down unit. Such practice misses the opportunity to provide this potentially life-saving intervention to babies who would benefit from and could be safely cared for using KMC practices. Current WHO guidelines recommend KMC for “stabilized” babies <2000g in hospital, but does not define “stabilized.” We contend that in many low-resource facility settings, small babies may be safely cared for using KMC practices while receiving oxygen, intravenous antibiotics, and some advanced care, therefore capturing the time of optimal benefit to save their lives through improved thermal care and breastfeeding. If KMC is conceptualized and operationalized as a care practice in conjunction with other care that may be required for the preterm newborn (eg, intravenous antibiotics, oxygen, feeding assistance) scaling up will save many lives. But if we fall into the trap of scaling up KMC as a place in a facility, far fewer lives will be saved, and we may thus have contributed to “empty scale-up” of KMC [5].

**Figure:**
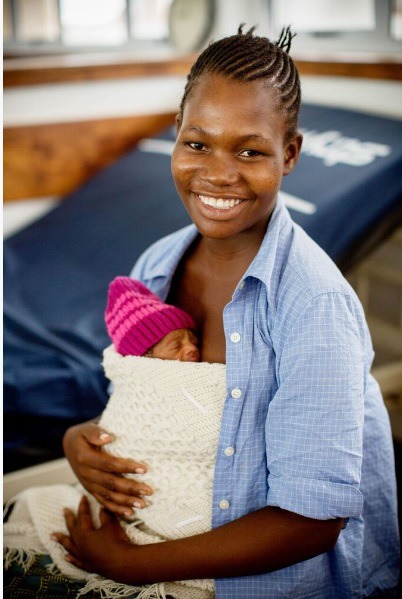
Photo credit: Save the Children (used with permission). Judith Gwiliza is a mother who recently gave birth to Jenney, her newborn baby. She had spent several days at the neonatal ward at Queen Elizabeth Hospital, Malawi. She was being taught the KMC (Kangaroo Mother Care) method to use on her baby.

KMC scale-up can be meaningful and impactful, if we pay attention to ensuring provision of the care practices that comprise KMC – namely prolonged, continuous skin-to-skin care (between the mother or other caregiver and the infant), and exclusive feeding with breast milk – rather than merely focusing on increasing the number of KMC units or spaces. And, while the principles of care for KMC can benefit all newborns, the use of continuous or intermittent KMC, integrated with other care needed by the preterm newborn, can be a matter of life or death for preterm infants. Ongoing research is addressing whether clinically unstable preterm infants can also benefit from KMC, potentially increasing the impact of KMC by reaching the sickest babies. In any case, KMC should be integrated into newborn units, rather than only taking place in a separate unit, so that vulnerable preterm infants can receive KMC, oxygen for respiratory distress syndrome, and intravenous antibiotics at the same time, if needed. This will require KMC trainings that focus on skills and confidence of health care providers to monitor and care for sick premature infants in KMC position, as well as to empower and transition better care to the parent; innovative space solutions to accommodate mothers and family in newborn units; and augmented performance monitoring and management that goes beyond counting KMC inputs or number of KMC units.

To be sure, step-down KMC units may have a role to play as a space for mother-baby dyads following discharge from the newborn unit, in settings where frequent post-discharge follow-up visits may be difficult. But this must be in addition to KMC received in the newborn unit, while preterm infants are most vulnerable. To ensure scale-up of effective KMC for the babies that need it most, we must be careful that we are scaling up the right thing.
